# Multiyear Wastewater Monitoring of High-Risk Substances Surrounding an Annual Electronic Dance Music Festival, Las Vegas, Nevada, USA, 2023-2025: Surveillance Study

**DOI:** 10.2196/102679

**Published:** 2026-09-08

**Authors:** Daniel Gerrity, Casey A Barber, Rebecca A Trenholm, Brett J Vanderford, Katherine C Crank, Ching-Lan Chang, Michael A Moshi, Anil T Mangla, Cassius Lockett, Edwin C Oh

**Affiliations:** 1 Applied Research and Development Center, Southern Nevada Water Authority, Las Vegas, NV, United States; 2 Department of Civil and Environmental Engineering and Construction, Howard R. Hughes College of Engineering, University of Nevada, Las Vegas, 4505 S Maryland Pkwy, Mail Stop 4005, Las Vegas, NV, 89154, United States, 1 702 856 3518; 3 Laboratory of Neurogenetics and Precision Medicine, College of Sciences, University of Nevada, Las Vegas, Las Vegas, NV, United States; 4 Neuroscience Interdisciplinary Ph.D. Program, University of Nevada, Las Vegas, NV, United States; 5 Center for Water Intelligence and Community Health, University of Nevada, Las Vegas, Las Vegas, NV, United States; 6 Southern Nevada Health District, Las Vegas, NV, United States; 7 School of Public Health, University of Nevada, Las Vegas, NV, United States; 8 Department of Brain Health, School of Integrated Health Sciences, University of Nevada, Las Vegas, Las Vegas, NV, United States; 9 Department of Internal Medicine, Kirk Kerkorian School of Medicine, University of Nevada, Las Vegas, Las Vegas, NV, United States

**Keywords:** high-risk substances, HRS, opioid, 3,4-methylenedioxymethamphetamine, MDMA, wastewater monitoring, event-based wastewater surveillance, wastewater-based epidemiology, WBE, substance use prevention, public health

## Abstract

**Background:**

Prior studies involving music festivals (including on-site surveys, drug checking, and wastewater-based analyses) suggest that recreational substance use is common among festivalgoers, though preferred substances and usage may be region- and event-specific. However, multiyear, event-specific wastewater surveillance data in the United States are limited.

**Objective:**

This study aimed to characterize concentrations of high-risk substances (HRS) and their metabolites in wastewater surrounding an annual electronic dance music (EDM) festival in Las Vegas, Nevada, USA. Using a progressive sampling approach over 3 consecutive years (2023‐2025), we assessed trends and examined comparisons by sample type, day, and location to inform situational awareness and future public health surveillance efforts and risk mitigation strategies.

**Methods:**

Wastewater samples were collected from utility access points at the festival site (2024‐2025), off-site in the surrounding community (2023‐2024), and at the downstream wastewater treatment plant (WWTP; 2023‐2025) during the festival week and weekend. For comparison, samples from a nonfestival weekend were collected off-site and at the WWTP in 2023. Target analytes consisted of 24 parent compounds and metabolites, including stimulants, opioids, and sedatives, which were analyzed by liquid chromatography–tandem mass spectrometry (LC-MS/MS) with isotope dilution. Analyte selection prioritized substances associated with overdose risk and with prior festival-related surveillance.

**Results:**

Cocaine (and 3 of 4 measured metabolites), methamphetamine, amphetamine, 11-nor-9-carboxy-Δ-9-tetrahydrocannabinol (THC-COOH), and tramadol were detected in 100% of festival weekend samples; 3,4-methylenedioxymethamphetamine (MDMA; ecstasy) was detected (at concentrations of up to nearly 2 mg/L) in all but 1 festival site sample collected prior to the start of the event in 2025. Festival-associated signals were most pronounced for stimulant-related analytes, which were also detected in WWTP and off-site samples during the festival. Compared with nonfestival weeks, MDMA concentrations were approximately 10 to 30 times higher at off-site locations and nearly 100 times higher at the downstream WWTP during the festival. Opioid-related concentrations were comparable at the WWTP and the festival site, although there were notable detections of norfentanyl and relatively high concentrations of tramadol in some festival site samples. MDMA- and cocaine-related analyte concentrations peaked during the final festival days in 2024 and 2025; the 2023 samples highlighted the presence of HRS in local wastewater during nonfestival weekends.

**Conclusions:**

Event-focused, multiyear wastewater surveillance detected stimulants and psychoactive substances, opioids, and Δ-9-tetrahydrocannabinol (THC) associated with an annual EDM festival weekend. Findings are consistent with previous wastewater-based studies of music and dance festivals, with MDMA concentrations among the highest reported in similar surveillance efforts. Codetection of opioid- and stimulant-related analytes suggests varied usage preferences, polysubstance use, and/or unintentional adulteration (eg, lacing). These findings demonstrate the value of wastewater monitoring as a complementary, population-level tool to support public health preparedness, harm-reduction strategies, and attendee safety during large-scale events. These data also inform wastewater utilities about expected influent composition during special events, with relevance for treatment operations and water reuse applications.

## Introduction

Large events, including music festivals, can present unique public health and safety challenges, particularly if attendees choose to engage in high-risk health behaviors such as recreational substance use. Surveys suggest that substance use is common among festivalgoers [1,2], with 24% of respondents in one study reporting an intention to use drugs at music festivals [3]. Researchers have previously investigated underlying reasons for adverse outcomes associated with festival-specific substance use behaviors, identifying a complex blend of factors such as first-time use among inexperienced attendees, new or unfamiliar drug sources (ie, drugs purchased from strangers), crowding, heat, exhaustion, and more [4]. The presence of adulterated or counterfeit drugs in this setting further exacerbates potential health risks among festivalgoers; a review of 6 studies involving voluntary drug-checking stations at festival sites found adulterants in 11% to 55% of tested samples [5]. These data sources may still underestimate the scope of this problem, as most event-related surveys, interviews, and drug-checking studies rely on voluntary participation and self-reporting of stigmatized (and often illicit) substance use behaviors.

In the search for a complementary, noninvasive tool for characterizing event-related recreational substance use, researchers have explored wastewater monitoring and wastewater-based epidemiology (WBE) of high-risk substances (HRS) at music festivals. Wastewater results from festivals in Australia [6,7], Brazil [8], and 6 European countries [9] suggest event- and region-specific differences in the types and amounts of drugs present. Lai et al [6] noted consistency in per capita consumption estimates for conventional and emerging illicit drugs (based on daily, on-site, and composite wastewater samples) across 2 years of a recurring summer music festival in Australia, suggesting that attendees may exhibit consistent year-over-year substance use patterns surrounding an event.

Las Vegas, Nevada, USA, is an international entertainment destination with multiple large annual events. Year-round activities draw tourists and locals alike, but one of the largest annual events is an electronic dance music (EDM) festival that has attracted more than 520,000 annual attendees in recent years [10]. During the festival weekend in 2022, surface water in the Las Vegas Wash, which primarily conveys discharged effluent from local wastewater treatment plants (WWTPs), was analyzed for the presence of pharmaceuticals and recreational drugs that had persisted through wastewater treatment, as a means of assessing ecological and environmental risks [11]. That study identified a unique fingerprint of community substance use associated with the festival weekend, including 3,4-methylenedioxymethamphetamine (MDMA; ecstasy), methamphetamine, cocaine, and ketamine. In fact, the median MDMA concentration in the Las Vegas Wash during the festival was 46 times higher than the study control (ie, nonfestival period) [11]. Elevated concentrations of drugs and their metabolites have important implications for Las Vegas Wash ecology and for understanding substance use in the local community during the event. However, due to the efficacy of wastewater treatment in attenuating concentrations of some trace organics [12,13], including HRS [14], direct monitoring of untreated wastewater (rather than effluent-impacted surface water) is preferable for WBE efforts.

Building on these local findings [11], this study characterizes HRS occurrence in site-specific and community-level raw wastewater surrounding the festival weekend over 3 consecutive years (2023, 2024, and 2025). Specifically, this study examines wastewater-based assessments of HRS over time and across sampling locations, festival days, and sample types (grab vs composite). This work further characterizes the wastewater-based HRS profile of the festival weekend for both the corresponding wastewater utility (to understand event-related changes in influent wastewater composition and to potentially inform wastewater treatment and reuse operations) and public health and safety personnel (to inform future event-related public health surveillance activities, responses, and resource allocations).

## Methods

### Festival Information

During each year of the study period (2023‐2025), the festival was held over a single mid-May weekend at a large outdoor venue within the same sewershed as the casino-resort corridor known as the Las Vegas Strip. Off-site promotional events catering to attendees began as early as the preceding Tuesday, but the on-site campground opened on Thursday. The on-site festival was then held nightly on Friday, Saturday, and Sunday, closing at 5:30 AM each morning. The festival concluded on Monday morning, with the campground closing later that day.

### Wastewater Sampling

#### Sampling Locations and Sample Handling

**Figure 1.**
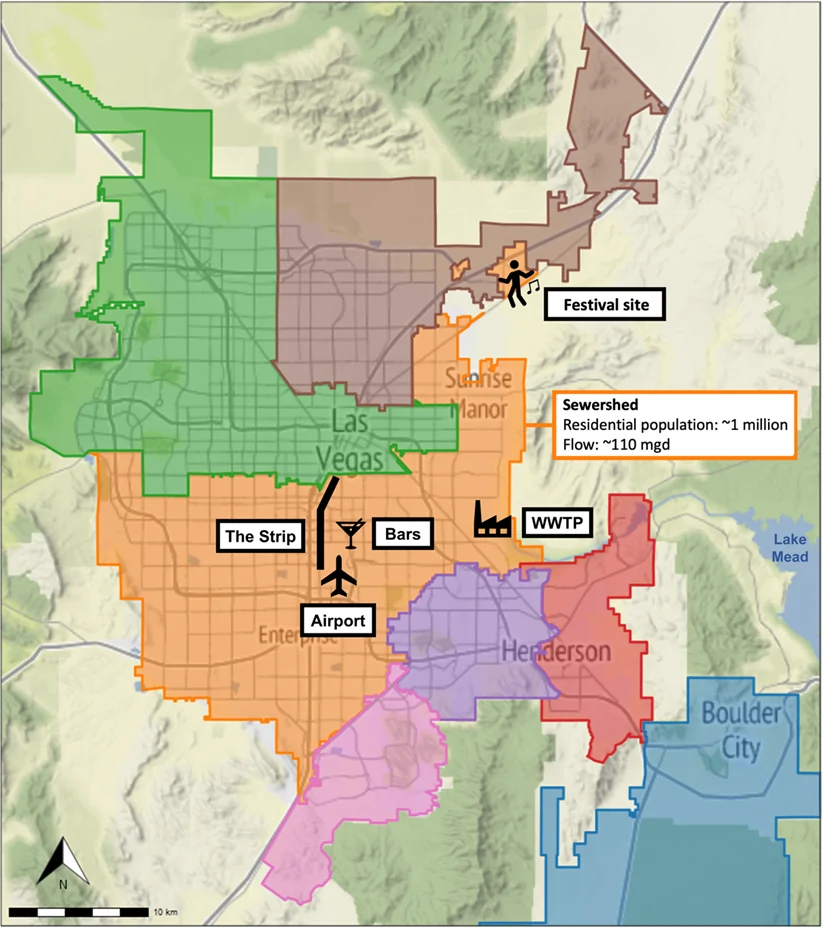
Map of the Las Vegas area sewersheds and wastewater sampling locations. mgd: million gallons per day; WWTP: wastewater treatment plant.

Festival-related wastewater sampling expanded each year of the study, from pilot sampling in 2023 to more comprehensive sampling before, during, and after the festival weekend in 2024 and 2025. Similar sampling schemes (pre-event, during, and postevent) have been described in previous event-based wastewater monitoring studies [15]. Sample locations and types by year and collection day are shown in Table 1. An aliquot (80 mL) of each collected sample was transferred to two 40-mL amber glass vials (containing 50 mg/L of ascorbic acid for oxidant quenching and 1 g/L of sodium azide for biological preservation) and then stored at 4°C until processing and analysis. A map of these sampling locations in the context of Las Vegas area sewersheds is shown in Figure 1. Although the site-specific sampling efforts required mapping of wastewater flows originating from contributing buildings, sites, or complexes, it was not possible to determine each sample’s specific points of origin *within* those contributing buildings, sites, or complexes, thereby maintaining individual privacy.

**Table 1.** Wastewater sampling locations by day and year during the festival week and weekend. Samples were composite unless otherwise noted^a^.

Year	Festival week and weekend sample collection days					
	Thursday	Friday	Saturday	Sunday	Monday	Tuesday
2023^b^	—^c^	—	Strip	Bars	WWTP^d^^,^^e^ Airport	—
2024	—	Festival^d^ WWTP Airport	Festival^d^ WWTP Strip Bars	Festival^d^ WWTP	Festival^d^ WWTP (n=2)^f^	—
2025	Festival WWTP	Festival WWTP	Festival WWTP	Festival WWTP	Festival WWTP	Festival (n=2)^f^ WWTP (n=2)^f^

a Off-site sample locations include airport, bars, and Las Vegas Strip.

b Additional nonfestival-weekend samples from the Las Vegas Strip, bars, airport, and WWTP (n=1 per site; not shown here) were collected in June and July 2023 for comparison.

c No samples collected.

d Grab sample only.

e WWTP: wastewater treatment plant.

f One composite and one grab sample were collected for sample-type comparison.

#### WWTP Sampling

Raw influent wastewater samples were collected each year from the WWTP downstream of the event venue by collaborating utility partners. This WWTP treats approximately 110 million gallons per day (mgd) and serves the casino-resort corridor of the Las Vegas Strip (with an average weekly visitor volume of approximately 800,000 throughout the year), in addition to the international airport, a large university, and a residential population of approximately 1 million. In 2023, 1 grab sample was collected at the WWTP after the final night of the festival (on Monday morning, May 22, 2023), and again 5 and 7 weeks later (nonfestival Mondays of June 26 and July 10, 2023, respectively). In 2024, 24-hour volume-proportional (flow-paced) influent composite samples were collected each morning during the festival (starting Friday and ending Monday, with collections representing the preceding 24 h), with a corresponding grab sample collected for comparison on Monday morning (as in 2023). Finally, the 2025 WWTP sampling expanded the 2024 sampling scheme to include composite sampling on Thursday (prefestival) and Tuesday mornings (postfestival), with another grab sample collected for comparison on Tuesday.

#### Community (Off-Site) Sampling

Time-proportional composite samples were collected using an autosampler (AS950, Hach) from utility access points (ie, manholes) receiving wastewater from the international airport, the casino-resort corridor of the Las Vegas Strip, and a cluster of bars and nightclubs near the Strip. Sampling details associated with these “off-site” manhole locations were described previously [16], and the days of the week represented by these samples are detailed in Table 1 and Multimedia Appendix 1. In 2023, samples from the airport, the Las Vegas Strip, and bars were collected during the festival weekend (in May) and the 2 nonfestival comparison weekends (in late June and early July). Similar sentinel-site sampling was performed in 2024 but not in 2025. Previous event-based HRS wastewater studies similarly included samples from the surrounding community [6] and nonfestival weekends [9] for comparison. No flow data were available for these sampling locations.

#### Festival-Site Sampling

In response to preliminary community-scale results from 2023, festival site-specific sampling was initiated in 2024 and repeated in 2025. Wastewater was collected by the collaborating wastewater utility from a manhole receiving wastewater only from the festival venue and campground (ie, mobile showers and restrooms discharging to the sewer collection system); these flows ultimately travel to the downstream WWTP. In 2024, a field-deployable autosampler was not yet available for use by the collaborating utility, so grab samples were collected daily from Friday to Monday morning (n=4) to complement the samples collected at the downstream WWTP. In 2025, the sampling period was extended from Thursday (prefestival) to Tuesday (postfestival), and, in lieu of grab samples (except for a supplemental Tuesday grab), 24-hour time-proportional composite samples were collected using an autosampler (CA101, C.E.C. Analytics). Therefore, sample type is a potential confounding variable for direct comparisons between 2024 and 2025 concentrations. The festival attracted more than 520,000 attendees each year, but no corresponding wastewater flow data were available for the festival-site sampling.

### Sample Analysis

Target analytes in this study included 24 parent compounds and metabolites (Table S1 in Multimedia Appendix 2). As in our concurrent campus-based wastewater monitoring campaign [17], nonopioid substances included cocaine and its metabolites (benzoylecgonine [BZE], ecgonine methyl ester [EME], ecgonine, and norcocaine), methamphetamine (and amphetamine), MDMA (and 3,4-methylenedioxyamphetamine [MDA]), Δ-9-tetrahydrocannabinol (THC; also 11-hydroxy-Δ-9-tetrahydrocannabinol [THC-OH] and 11-nor-9-carboxy-Δ-9-tetrahydrocannabinol [THC-COOH]), and the tranquilizer xylazine. Natural (heroin, morphine, and codeine), semisynthetic (hydrocodone and oxycodone), and synthetic (fentanyl, methadone, and tramadol) opioid targets were also included. Relevant opioid metabolites included 6-acetylmorphine for heroin, norfentanyl for fentanyl, and 2-ethylidene-1,5-dimethyl-3,3-diphenylpyrrolidine (EDDP) for methadone. Fentanyl and xylazine were added to the analyte panel in 2025.

Analyses involved direct injection of aqueous samples after a 10-fold dilution to reduce matrix interference; samples were diluted further if concentrations fell outside the calibration range. All compounds were analyzed by liquid chromatography–tandem mass spectrometry (LC-MS/MS) with isotope dilution quantification according to previously published methods [18,19]. Analyses used a CTC Autosampler (CTC Analytics) and a 1260 LC Binary Pump (Agilent). Target compounds and their isotopically labeled analogs were then analyzed using positive electrospray ionization (ESI) in multiple reaction monitoring (MRM) mode on a 6500 QTRAP mass spectrometer (SCIEX). Additional analytical details for all target compounds, including method reporting limits (MRLs) and MRM transitions, are included in Text S1 in Multimedia Appendix 2. Field and analytical blanks (consisting of Milli-Q water treated as samples) were negative for all target compounds.

### Statistical Analysis

Wilcoxon signed-rank tests were conducted in R using RStudio. The first analysis compared site-specific analyte concentrations between 2024 and 2025 (ie, festival 2024 vs festival 2025 and WWTP 2024 vs WWTP 2025), and data were limited to sampling days that overlapped in both years. Note that the festival comparison was potentially confounded by the difference in sample type (grab in 2024 and composite in 2025). The second analysis compared same-day concentrations at the festival site and the WWTP across both 2024 and 2025. Any concentrations below the MRL were omitted from the statistical analyses, along with their paired samples.

### Ethical Considerations

The University of Nevada, Las Vegas Institutional Review Board reviewed this project and determined it to be exempt from human participant research regulations and from the requirement for informed consent, according to federal regulations and university policy.

## Results

### Detection Frequencies and Analyte Concentrations by Year and Sampling Location

#### Principal Findings

Analyte detection frequencies and corresponding concentrations were shared electronically with the collaborating wastewater utility and the local health authority as soon as the data were finalized. Detection frequencies for each analyte by year and sampling location during the festival weekend are shown in Table 2, with the airport, bars, and Las Vegas Strip sampling sites combined as “off-site” locations.

Cocaine (and 3 of 4 metabolites), methamphetamine, amphetamine, THC-COOH, and tramadol were detected (above the MRL) in 100% of samples across all years and all locations during the festival week and weekend (excluding the 2023 nonfestival comparison weeks). This finding is consistent with previous wastewater surveillance studies in Southern Nevada; for example, methamphetamine and THC typically exhibit some of the highest per capita consumption rates based on Southern Nevada’s community-scale wastewater concentrations [19]. Additionally, amphetamine has been one of the most frequently detected compounds in the sewer collection system of a local university campus [17]. In 2010, elevated cocaine consumption was linked to a major sporting event weekend based on wastewater samples collected from the same Southern Nevada WWTP in this study [14]—a finding also observed in 2023 [19]. Here, norcocaine—the one exception among the cocaine metabolites—was detected in only a subset of the samples collected upstream of the WWTP, specifically in all 3 samples from the bars in 2023 (festival and nonfestival weekends) and in 1 sample from the festival site in 2025 (grab sample only). Neither heroin, THC, nor xylazine (analyzed only in 2025) was detected above the MRL in any samples in this study, and the major heroin metabolite acetylmorphine was not detected at all in 2025 or at the WWTP in 2024.

MDMA was detected in all festival-impacted samples (including off-site, festival site, and WWTP samples), except for a 2025 festival site composite collected *before* the campground was officially opened for the weekend. In the previous community-scale study, MDMA occurrence was rare in neighboring Southern Nevada sewersheds (20%±8% detection frequencies in the previous study), compared to the WWTP in this study (92% detection frequency previously) [19]. MDMA occurrence in influent wastewater at this WWTP is presumably driven primarily by contributions from the Las Vegas Strip, but the high MDMA detection frequency across all festival weekend samples (including those from the airport) highlights the impact of the event across the city. Although detected in 100% of festival weekend off-site samples, MDMA was below the MRL in both airport samples and in one of the Las Vegas Strip samples during nonfestival weeks in 2023, and it was also below the MRL in all samples collected at a local university campus in 2025 [17]. Similarly, MDA (a major metabolite of MDMA) is rarely observed in local wastewater samples; for example, the only detection of MDA in the previous sewershed-level wastewater study in Southern Nevada was associated with an unrelated music festival in September 2022 [19]. Thus, the MDA detection frequencies in this study, primarily associated with the bars and festival samples, are notable.

Although stimulant-related analytes were most frequently detected, several opioid-related analytes (eg, tramadol, codeine, hydrocodone, oxycodone, norfentanyl, and morphine) were present in more than 50% of samples across the study years and locations. Some detection frequencies varied by location; for example, codeine, hydrocodone, and norfentanyl detection frequencies were lower at the festival site compared with off-site (2024) and the WWTP (2024 and 2025). Both festival and off-site samples yielded fewer detections of EDDP compared to the WWTP samples. The co-occurrence of opioids and stimulants may reflect a variety of substance use behaviors, preferences, polysubstance use, and/or unintentional adulteration (ie, lacing).

Analyte concentrations by year and sample location during the festival week and weekend are shown in Multimedia Appendix 2 (Figure S1), and concentrations are listed in Multimedia Appendix 1. Within each site, and despite the difference in sample type at the festival site (ie, grab in 2024 and composite in 2025), concentrations of each analyte were not significantly different between 2024 and 2025 (all Wilcoxon signed-rank *P*>.05). Notably, several stimulant-related (cocaine metabolites, amphetamine, methamphetamine, MDA, and MDMA) and THC-related (THC-COOH and THC-OH) concentrations observed in the festival site wastewater exceeded those in samples from the WWTP or off-site locations by more than an order of magnitude (Figure S1, Multimedia Appendix 2). In fact, stimulant-related analytes had some of the highest observed wastewater concentrations, with BZE reaching as high as 587,000 ng/L at the festival site. This was exceeded only by MDMA in festival site samples, which reached 1,280,000 ng/L in 2024 and 1,860,000 ng/L in 2025; these concentrations are among the highest festival-related wastewater MDMA concentrations reported in the literature.

**Table 2.** Detection frequency (%) across all samples (grab and composite) by year and location during the festival week and weekend^a^.

Analyte	Festival week and weekend detection frequencies (% of N samples above MRL^b^)						
	2023: sample locations; sample types		2024: sample locations; sample types			2025: sample locations; sample types	
	Off-site^c^ (N=3); composite (n=3), %	WWTP^d^ (N=1); grab (n=1), %	Off-site^c^ (N=3); composite (n=3), %	Festival (N=4); grab (n=4), %	WWTP (N=5); composite (n=4) and grab (n=1), %	Festival (N=7); composite (n=6) and grab (n=1), %	WWTP (N=7); composite (n=6) and grab (n=1), %
Cocaine	100	100	100	100	100	100	100
BZE^e^^,^^f^	100	100	100	100	100	100	100
Ecgonine^e^	100	100	100	100	100	100	100
EME^e^^,^^g^	100	100	100	100	100	100	100
Norcocaine^e^	33	0	0	0	0	14	0
Codeine	100	100	100	75	100	57	100
Hydrocodone	100	100	100	75	100	29	0
Oxycodone	100	100	100	100	100	86	100
Fentanyl^h^	—^i^	—	—	—	—	0	14
Norfentanyl^e^	100	100	100	75	100	43	100
Heroin	0	0	0	0	0	0	0
Acetylmorphine^e^	67	100	67	50	0	0	0
MDMA^j^	100	100	100	100	100	86	100
MDA^k^	100	100	100	100	80	71	57
Methadone	0	100	33	0	0	14	0
EDDP^e^^,^^l^	33	100	67	25	100	43	100
Methamphetamine	100	100	100	100	100	100	100
Amphetamine	100	100	100	100	100	100	100
Morphine	100	100	100	100	100	86	100
THC^m^	0	0	0	0	0	0	0
THC-COOH^e^^,^^n^	100	100	100	100	100	100	100
THC-OH^e^	0	100	0	100	0	86	29
Tramadol	100	100	100	100	100	100	100
Xylazine^h^	—	—	—	—	—	0	0

a An analyte was “detected” if its concentration was above the MRL.

b MRL: method reporting limit.

c Off-site locations in 2023‐2024 are airport, bars, and Las Vegas Strip. The 2023 nonfestival-weekend samples are excluded here.

d WWTP: wastewater treatment plant.

e These analytes are metabolites only.

f BZE: benzoylecgonine.

g EME: ecgonine methyl ester.

h Samples were analyzed for fentanyl and xylazine in 2025 only.

i Not available.

j MDMA: 3,4‑methylenedioxymethamphetamine.

k MDA: 3,4-methylenedioxyamphetamine.

l EDDP: 2-ethylidene-1,5-dimethyl-3,3-diphenylpyrrolidine.

m THC: tetrahydrocannabinol.

n THC-COOH: 11-nor-9-carboxy-Δ-9-tetrahydrocannabinol.

#### 2023 Pilot Year: Festival vs Nonfestival Weeks

Analyte concentrations in the 2023 samples are shown in Multimedia Appendix 2 (Figure S2) by sample location and collection event (festival weekend in May vs nonfestival weekends in June and July). Although no 2023 sampling occurred at the festival site, MDMA and MDA concentrations were notably higher in the festival weekend samples across all sites (bars, the Las Vegas Strip, the airport, and the WWTP). In fact, the airport-related detections of MDMA and MDA occurred only during the festival weekend, highlighting potential contributions from visitors traveling to Las Vegas for festival-related activities. MDMA concentrations at the bars and the Las Vegas Strip were approximately 10 and 30 times higher, respectively, during the festival compared with nonfestival sampling events, and nearly 100 times higher at the WWTP (treating relatively consistent community flows of ~110 mgd). Along with the approximate 10-fold change in wastewater MDMA concentration at the bars, the norfentanyl concentration was nearly 2 times higher during the festival week, highlighting potential risks of fentanyl use, adulteration, or contamination [20], and the need for increased awareness of these possibilities [21].

Concentrations of other substances (eg, methamphetamine, amphetamine, and THC metabolites) were similar or even higher on nonfestival weekends; a similar observation for methamphetamine was observed for a baseline (higher concentration) vs special event weekend (lower concentration) in an earlier study conducted in Las Vegas [14]. Regardless of the week, wastewater concentrations at the bars were consistently higher than those at the Las Vegas Strip, the airport, and the WWTP for cocaine (and multiple metabolites) and methamphetamine. Importantly, these results highlight the widespread consumption of HRS across Southern Nevada during festival and nonfestival weekends.

#### 2024-2025: Day of Festival Week

**Figure 2.**
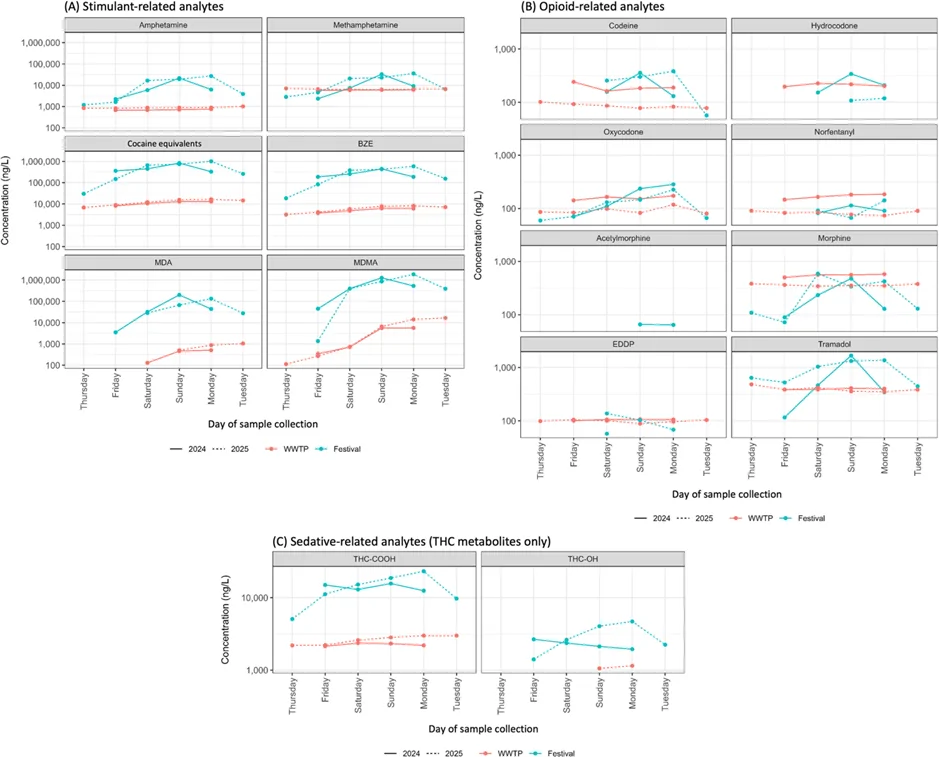
Concentrations in wastewater from the festival site (blue) and WWTP (red) by day of collection in 2024 (solid line) and 2025 (dashed line) for (A) stimulants, (B) opioids, and (C) sedatives (THC-COOH and THC-OH only). Note: 2024 festival samples are grab samples; others are composite. Analytes not detected in either year (heroin, THC, and xylazine) and analytes with singular detections (fentanyl, methadone, and norcocaine) are not shown. BZE: benzoylecgonine; EDDP: 2-ethylidene-1,5-dimethyl-3,3-diphenylpyrrolidine; MDA: 3,4-methylenedioxyamphetamine; MDMA: 3,4‑methylenedioxy‑methamphetamine; THC: tetrahydrocannabinol; THC-COOH: 11-nor-9-carboxy-Δ-9-tetrahydrocannabinol; WWTP: wastewater treatment plant.

Analyte concentrations by year and day of wastewater sample collection (Friday-Monday in 2024; Thursday-Tuesday in 2025) and sample location (festival vs WWTP) are shown in Figure 2; analytes not detected at these sites in either year (heroin, THC, and xylazine) and analytes with singular detections (fentanyl, methadone, and norcocaine) are not shown. Gerrity et al [19] previously documented the instability of cocaine-related compounds in wastewater but demonstrated that the overall mass balance of cocaine and its metabolites was conserved over 60 days of hold time. Thus, the cocaine-related compounds are aggregated in Figure 2 as “cocaine equivalents” (after accounting for differences in molecular weight), with BZE also shown for comparison.

For the analytes detected at least twice, concentrations tended to peak in samples collected on either Sunday or Monday, surrounding the culmination of the festival. Increasing concentrations of stimulants (including amphetamine, methamphetamine, MDMA, MDA, BZE, and cocaine equivalents in Figure 2A) were particularly evident over the main festival days at both sites. In 2025, festival-site MDMA concentrations increased rapidly from below the MRL before the campground was opened to 1370 ng/L after the first full day of the festival, and ultimately peaked at 1,860,000 ng/L at the end of the weekend (in the Sunday-Monday sample); residual high concentrations persisted through the Tuesday sample collections.

Although each analyte’s concentration within the sampling sites did not differ significantly between 2024 and 2025, concentrations across both years were significantly different between the festival site and the WWTP for amphetamine, cocaine equivalents, BZE, ecgonine, MDMA, and THC-COOH (all Wilcoxon signed-rank *P*=.008). As expected due to significant dilution from municipal wastewater flows, stimulant concentrations were often lower at the WWTP relative to the festival site. That being said, BZE more than doubled, and MDMA increased more than 100-fold at the WWTP during the 2025 festival week. THC-COOH and THC-OH were also higher in the festival samples compared to the WWTP samples (Figure 2C). Although there were some day-to-day variations, opioid-related concentrations were generally more similar or even higher at the WWTP relative to the festival (Figure 2B), but some opioids (eg, oxycodone, morphine, and tramadol) exhibited increasing trends at the festival site over the first few days of the festival.

#### 2024-2025: Grab vs Composite Sampling

**Figure 3.**
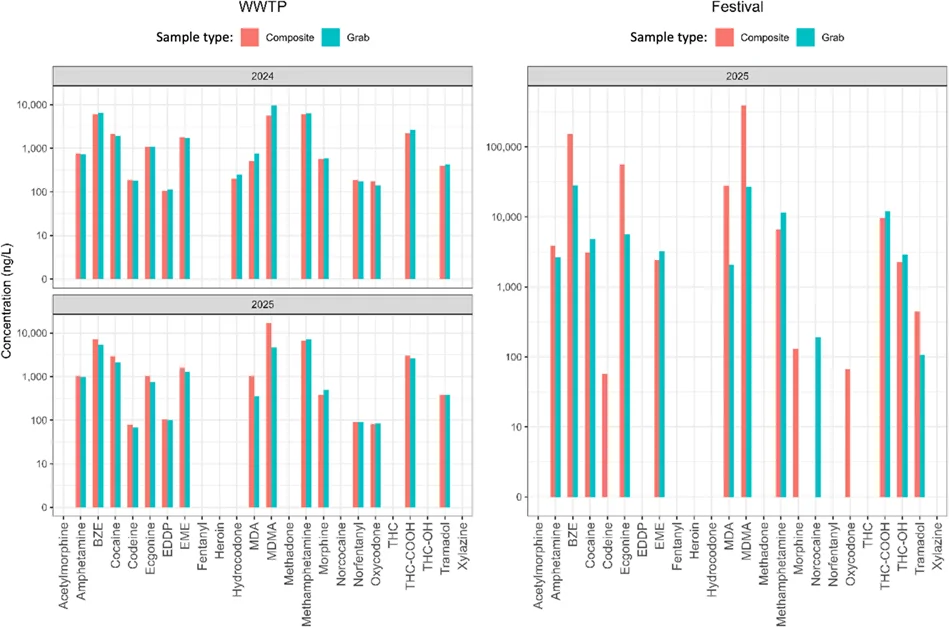
Comparison of analyte concentrations in corresponding composite (red) and grab (blue) samples for the WWTP (2024 and 2025; left) and the festival site (2025 only; right). BZE: benzoylecgonine; EDDP: 2-ethylidene-1,5-dimethyl-3,3-diphenylpyrrolidine; EME: ecgonine methyl ester; MDA: 3,4-methylenedioxyamphetamine; MDMA: 3,4‑methylenedioxy‑methamphetamine; THC: tetrahydrocannabinol; WWTP: wastewater treatment plant.

As shown in Figure 3, concentrations were similar between grab and composite samples collected at the WWTP in 2024. In 2025, WWTP composite sample concentrations were higher than the corresponding grab samples for the analytes expected to be impacted by the festival, including some cocaine-related compounds, MDA, and MDMA. These differences between 2024 (Monday collections) and 2025 (Tuesday collections) may have been driven by the day of the week, with the Tuesday grab likely to be less affected by the festival weekend. Similarly, the 2025 festival composite sample (Monday-Tuesday) yielded higher concentrations of BZE (5.5-fold), ecgonine (9.9-fold), MDA (13.4-fold), MDMA (14.6-fold), and tramadol (4.2-fold) compared to the corresponding Tuesday grab sample. Notably, codeine, morphine, and oxycodone were detected in the composite but not in the corresponding grab sample, while only norcocaine was detected in the grab sample and not in the corresponding composite sample.

## Discussion

### Principal Findings

This multiyear surveillance study analyzed wastewater from multiple locations to characterize the presence and concentration of substances (and their metabolites) surrounding an annual EDM festival. The findings in 2023 reaffirmed the presence of HRS in raw community wastewater during festival and nonfestival time points, with notable differences by weekends and sample locations. The findings from 2024 to 2025 further illustrated the prevalence of stimulants (cocaine, methamphetamine, and notably MDMA) in festival-site wastewater, although opioid-related analytes were also detected there and in the community. Detections related to THC were not unexpected, given the legality of recreational marijuana products in the state of Nevada and prior literature indicating its frequency in festival-related WBE studies [22].

The study festival (with more than 520,000 annual attendees) is large in comparison to other events included in prior wastewater-based studies of substance use. The 2010 to 2011 Australian festival described by Lai et al [6], for example, had daily attendees between 12,100 and 19,700; Bijlsma et al [9] sampled from 6 European music festivals with a total combined attendance of 465,000. A recent systematic review [22] included 23 WBE studies on HRS, with 6200 to 600,000 attendees; only 2 studies reported attendance in excess of 500,000 people.

Although this study included just 3 sequential years, the results offer a preliminary wastewater-based profile of substances and metabolites present at the festival site, namely stimulants (eg, MDMA, cocaine, and methamphetamine), as well as select opioids. Lai et al [6] similarly noted consistency in festival attendance and wastewater-based HRS profiles each year, in line with findings that event-related WBE findings may vary by attendee characteristics and time of year [23]. Elsewhere, researchers have documented wastewater-based concentrations rising to a peak during the event [23], a pattern also observed here on the final days of the festival and potentially reflecting accumulation and/or intentional discarding at the end of the event [8]. Multiple WBE studies have highlighted wastewater-based profiles of substance use surrounding holidays [23-25], during weekends compared to weekdays [24], and among nightclub, music event, and festival attendees in general [26-28], with some variation by region and music genre [22]. Several results in this study indicated concurrent detections of select analytes (eg, MDMA and norfentanyl at the bars), highlighting another potential opportunity for multifaceted public health responses to address intentional (or unintentional) polydrug use [29] amid the recent wave of polysubstance use and related deaths in the US overdose crisis [30].

### MDMA and Other Stimulants

Across analytical approaches, geographic locations, and event types, MDMA was the most consistently detected substance in the Cainamisir et al [22] review of 23 wastewater studies of psychoactive substances at music festivals. The maximum MDMA concentrations in this study (1,280,000 ng/L and 1,860,000 ng/L), however, exceeded concentrations reported previously [22], including those from a neighboring Southern Nevada sewershed during a separate music festival in the fall of 2022 (1100 ng/L) [19] and from a festival in Thailand (1267 ng/L) [31]. An analysis of pooled wastewater from a festival in Belgium, however, reported a higher concentration (7580 ng/mL=7,580,000 ng/L) [32]. The accumulation of MDMA over a festival weekend (with a blood half-life of 8 h; 95% clearance within 40 h [33]) may help explain the concentrations in this study. Importantly, prior work has observed that MDMA concentrations tend to be higher on weekends, even in the absence of a large event or festival [34,35]. Recent wastewater findings in Europe also emphasized higher MDMA loads during weekends compared to weekdays (occurring in more than 75% of cities studied), though overall MDMA wastewater loads in European cities decreased by nearly 16% from 2024 to 2025 [36].

Methamphetamine and cocaine (and its metabolites) were consistently present over the festival weekends (with 100% detection frequencies), but the 2023 nonfestival weekend concentrations suggest community use independent of the event. A separate local wastewater surveillance study conducted in 2022 to 2023 [19] similarly concluded that methamphetamine and cocaine were among the highest per capita consumption estimates across all Las Vegas sewersheds. Wastewater detections of cocaine (the parent compound) have been reported in similar festival-based studies [22], albeit with slightly lower concentrations (eg, a maximum of 8914 ng/L in Brazil [8] vs 10,000 ng/L in this study). Here, we used a mass-balance approach to visualize total cocaine equivalents, but focusing on one of the more abundant cocaine metabolites, specifically BZE, may be sufficient for public health and event-related wastewater monitoring of cocaine use (Figure 2). Cainamisir et al [22] highlighted geographic variability in wastewater-based indicators of methamphetamine presence and consumption at festivals elsewhere, though the methamphetamine detections in this study align with the established presence of the substance in Southern Nevada [37].

### Opioid HRS Targets

Although stimulants were detected most frequently in this study, several opioids were also present during the festival week and weekend, both on-site and in the broader community. Norfentanyl, for example, was detected in both festival and WWTP wastewater, though more consistently at the WWTP; this aligns with the prior local findings [19] and could reflect some medical use. Other festival-related WBE studies have reported detections of norfentanyl, though it was not the most common analyte; in one study, norfentanyl was detected at only 2 of 6 festivals sampled [7]. Generally, festival-related opioid concentrations in this study mirrored those at the downstream WWTP, suggesting a less-pronounced event-associated use compared with the stimulants discussed earlier. However, the co-occurrence of some opioids and stimulants in site-specific wastewater samples may reflect intentional or unintentional polysubstance use (ie, through lacing) [29], although this cannot be confirmed with wastewater alone.

The absence of heroin (the parent compound) detections was not unexpected, given previous work suggesting its potential instability in the presence of the quenching agent, ascorbic acid, used here [19]. Heroin and its metabolite acetylmorphine are known to be subject to rapid degradation in wastewater [38], though acetylmorphine has been detected during previous hold-time experiments [19], in other local studies [17], and here in 2023 (n=4 off-site samples and 2 WWTP samples) and in 2024 (n=2 off-site samples and 2 festival-site samples).

### Limitations

Several elements of this study may limit and/or confound the interpretation of results and their broader contextualization in the fields of WBE and public health. First, due to practical and logistical limitations, the wastewater sampling framework varied each year of the study, complicating year-over-year comparisons. Multiday sampling in 2024 and 2025 offered more granular detail about the festival week and weekend, and the limited comparisons between grab and composite samples highlighted the potential effect of sample type on analyte detections (eg, codeine, morphine, oxycodone, and norcocaine) and on concentrations (eg, MDMA). Generally, composite samples are preferred over grab samples in the WBE literature, given concerns about potentially nonrepresentative slugs of wastewater that may underestimate or overestimate the presence of a target analyte in wastewater [39]. However, the results of this study show that even limited grab sampling offers valuable information about HRS presence and use surrounding an event.

Analyte source, consumption, metabolism, excretion, stability, and dilution in wastewater are also key factors that affect interpretation of wastewater results. Others have addressed metabolism with correction factors to back-calculate consumption estimates [19,40]. This would be a valuable addition to future festival-specific WBE studies for HRS, although corresponding flow measurements would be needed to calculate mass loadings. Wastewater analyses also do not distinguish between the various individuals present at a festival, potentially including attendees, artists, vendors, staff, and volunteers. Supplemental analyses to distinguish between licit and illicit consumption of select substances (eg, methamphetamine) have been established [41], but were not performed here. This means, for example, that a portion of the amphetamine and methamphetamine concentrations observed here may be due to medically prescribed use, rather than illicit use. Furthermore, these wastewater detections may partially reflect intentional discarding (ie, dumping) of substances in festival restrooms and subsequent in-sewer degradation during and/or at the end of the event, as has been noted previously [8]. Although wastewater monitoring does not identify specific sources (eg, original user or lacing vs polysubstance use vs intentional discarding), these findings still complement existing public health surveillance activities and indicate the presence of these substances on-site and in the community surrounding the event.

Results were not normalized to wastewater flow rate, a fecal indicator, or another analyte (eg, caffeine or sucralose). As described earlier, normalization would have facilitated consumption calculations and/or captured differences in human urine or fecal load within these samples [19]. Site-specific consumption estimates could be calculated with site-level flow data and corrections for metabolism or excretion or molecular weight equivalence [19,40]. Importantly, upstream flows may be highly variable throughout the day and across the event.

This study included a selection of conventional prescription and recreational substances of public health significance, but a variety of other targets could be included in future work. Several published event-focused studies have used wastewater to characterize the presence of less-regulated novel psychoactive substances (NPS) [7,9]. Wastewater may be particularly valuable in tracking the emergence of new substance use trends, including NPS and misuse of existing substances (eg, xylazine), in tandem with other sources such as online discussion forums [42]. Future wastewater-based efforts might also include medetomidine, naloxone (despite potential instability in wastewater [43]), and ketamine, which may be increasingly common [26] and co-occurring with fentanyl [44]. Others have previously characterized the community wastewater-based occurrence of ketamine in Europe [24]; ketamine was also found in the Las Vegas Wash during the 2022 festival weekend [11].

At least one similar WBE study endeavored to relate wastewater HRS results to clinical outcomes or other datasets, although these analyses were beyond the scope of this study. This previous festival-based work measured wastewater HRS concentrations along with self-reported substance use behaviors [7], but links between festival HRS wastewater data and health outcomes associated with substance use (eg, overdoses, health care encounters, and deaths) are less clear in the literature. This does not, however, diminish the serious health outcomes that have been reported among festivalgoers [4,45], including critical care admissions at a tertiary care hospital in Las Vegas associated with the festival weekend [46,47]. In a 2023 case series of intensive care unit (ICU) admissions, substances reportedly used included MDMA (6/6 patients), cocaine (n=2), fentanyl (n=1), lysergic acid diethylamide (LSD; n=1), THC (n=1), and/or alcohol [1,46]. A recent local retrospective cohort study also found that drug or alcohol overdose patients related to the festival in this study were younger and more likely to be admitted to the ICU than nonfestival-related drug or alcohol overdose patients, although the 2 groups did not differ significantly in other characteristics, drug use patterns, or ultimate health outcomes [47]. In 2025, the Clark County coroner’s office reported that MDMA contributed to 2 deaths in the Las Vegas area during the festival weekend [48].

### Implications for Public Health Response

These results offer valuable insights for public health and clinical situational awareness [22] of substance use during the festival week or weekend, complementing other surveillance activities, emergency responses, and the ultimate reporting of health care use and serious clinical outcomes. Selected results, such as the relatively few heroin-related detections in this study, may reflect an evolving landscape of substance use behaviors among attendees. Palamar et al [26] described changes in substances of choice in New York City nightlife and dance club settings from 2017 to 2022, with survey responses indicating decreased use of heroin, nonmedical prescription opioids, and cocaine (among other stimulants) in the post–COVID-19 era, accompanied by increased use of ketamine, psilocybin, poppers, and other NPS. Together with ongoing surveys, online forum monitoring [42], drug checking, and traditional public health or clinical surveillance, event-based wastewater surveillance may inform continued investment in prevention and future responses or interventions, both on-site and in the community, to support successful events while prioritizing attendee and community health and safety. Text S2 in Multimedia Appendix 2 provides additional discussion and recommendations on how event-based wastewater surveillance can inform on-site and community-level public health activities, including activities that have been implemented specifically for the music festival in question.

### Implications for Utilities

Event-related wastewater monitoring also offers insight into expected changes in the composition of influent wastewater flows surrounding the event. Although in-sewer mixing, dilution, and degradation likely explain the lower analyte concentrations in the WWTP samples compared with the festival site, the WWTP still experienced significant increases (10- to 100-fold) in influent concentrations of select analytes (eg, MDMA and MDA) over the event weekend. In addition to changes in flow driven by large influxes of people associated with special events, these compositional changes have implications for wastewater treatment processes, water reuse, and downstream environmental surface water quality [11,22,49], highlighting how wastewater monitoring can be informative and mutually beneficial for utilities and public health entities.

### Conclusions

Event-related wastewater monitoring is an evolving subtopic in the field of WBE. Several studies have already explored targeted wastewater monitoring for pathogens such as SARS-CoV-2 at the Olympics [50] and other mass gatherings [15], but WBE has also been applied to substance use at music festivals in countries like Australia and Thailand. In Las Vegas, this approach has now been extended to characterize the use of HRS at the community level and in treated wastewater effluent surrounding events and festivals, as has been done elsewhere [11,14,17,19]. Here, wastewater surveillance surrounding a large EDM festival in 3 sequential years at on-site and off-site locations further explored WBE as a complementary tool for public health monitoring of substance use or misuse. Stimulant concentrations were notably elevated during event weekends, with high peak concentrations of MDMA and cocaine-related analytes, but opioid-related detections were also observed. These findings may inform both public health surveillance and response activities and wastewater utility operations.

Wastewater monitoring should complement, not replace, existing public health surveillance and response structures. Multifaceted public health surveillance systems can integrate wastewater surveillance for both pathogens and HRS to expand event-related preparedness, including interventions to prioritize the health and safety of attendees on-site and in the community. Future research may explore additional HRS targets, other non-EDM festivals and events, trends over time, and alignment with other data sources (including health outcomes). Local public health agencies, community stakeholders (eg, health care systems, nonprofit organizations, and local government), and other tourism and event destinations similarly stand to benefit from data-driven understanding of the public health implications of events and mass gatherings.

## Supplementary material

10.2196/102679 Multimedia Appendix 1 Wastewater concentrations of analytes and corresponding sample information.

10.2196/102679 Multimedia Appendix 2 Supplemental analytical methods, compound and mass spectrometry parameters, wastewater concentration data, and additional discussion of on-site and community-level public health activities.

## Abbreviations

BZE: benzoylecgonine
EDDP: 2-ethylidene-1,5-dimethyl-3,3-diphenylpyrrolidine
EDM: electronic dance music
EME: ecgonine methyl ester
ESI: electrospray ionization
HRS: high-risk substances
ICU: intensive care unit
LC-MS/MS: liquid chromatography–tandem mass spectrometry
LSD: lysergic acid diethylamide
MDA: 3,4-methylenedioxyamphetamine
MDMA: 3,4‑methylenedioxy‑methamphetamine
MGD: million gallons per day
MRL: method reporting limit
MRM: multiple reaction monitoring
NPS: novel psychoactive substances
THC: Δ-9-tetrahydrocannabinol
THC-COOH: carboxy-Δ-9-tetrahydrocannabinol
WBE: wastewater-based epidemiology
WWTP: wastewater treatment plant
