# Supplementary material for: Multiyear Wastewater Monitoring of High-Risk Substances Surrounding an Annual Electronic Dance Music Festival, Las Vegas, Nevada, USA, 2023-2025: Surveillance Study
Source: JMIR Public Health Surveill. 2026 Sep 8;12:e102679. doi: 10.2196/102679 (PMC13552836; doi:10.2196/102679)
Supplement: Supplementary file 1 [file publichealth-v12-e102679-s002.pdf]

Multimedia Appendix (Supplementary Information)

Multi-year wastewater monitoring of high-risk substances surrounding an annual electronic dance music festival, Las Vegas, Nevada, USA, 2023-2025: Surveillance study

Daniel Gerrity[a,b,1*], Casey A. Barber[a,1], Rebecca A. Trenholm[a], Brett J. Vanderford[a], Katherine C. Crank[a], Ching-Lan Chang[c], Michael A. Moshi[c], Anil T. Mangla[d,e], Cassius Lockett[d], Edwin C. Oh[c,f]

[a] Applied Research and Development Center, Southern Nevada Water Authority, Las Vegas NV, USA

[b] Department of Civil & Environmental Engineering and Construction, University of Nevada, Las Vegas, Las Vegas, NV, USA

[c] Laboratory of Neurogenetics and Precision Medicine, College of Sciences; Neuroscience Interdisciplinary Ph.D. program; Center for Water Intelligence and Community Health, University of Nevada, Las Vegas, Las Vegas, NV, USA

[d] Southern Nevada Health District, Las Vegas, NV, USA

[e] School of Public Health, University of Nevada, Las Vegas, Las Vegas, NV, USA

[f] Department of Brain Health, School of Integrated Health Sciences; Department of Internal Medicine, Kirk Kerkorian School of Medicine, University of Nevada, Las Vegas, Las Vegas, NV, USA

[1] These first authors contributed equally to this article.

[*]**Corresponding Author:** Daniel Gerrity (daniel.gerrity@snwa.com; daniel.gerrity@unlv.edu; +1-702-856-3518; Southern Nevada Water Authority, P.O. Box 99954, Las Vegas NV, 89193; University of Nevada, Las Vegas, 4505 S Maryland Pkwy, Mail Stop 4005, Las Vegas, NV, 89154, USA

Text S1. Supplemental analytical methods.

The high-risk substances and metabolites listed in **Table S1** were analyzed in each wastewater sample by direct injection liquid chromatography tandem mass spectrometry (LC-MS/MS) after a 1:10 dilution (or greater when necessary) with reagent water. A 100 µL sample loop (sample volume) was used for each injection. Prior to injection, 50 µL of a 50-200 µg/L stock solution of isotopically labeled analogs (Cerilliant, Round Rock, TX, USA) was added to a 10 mL aliquot of each sample. This resulted in a final concentration of 0.25-1.0 µg/L of each isotope, which served as the basis for isotope dilution quantitation. Separation was performed on a 150 × 4.6 mm Raptor Biphenyl column with a 5 µm particle size (Restek, Bellefonte, PA, USA) at room temperature. LC mobile phases consisted of (A) 0.1% formic acid in reagent water and (B) 0.1% formic acid in methanol. The LC flow rate was set at a constant rate of 700 µL/min with a gradient as follows: initial = 5% B, at 0.5 min = 25% B, at 14 min = 100% B, hold until 22 min, followed by a 5 min equilibration at 5% B. The mass spectrometry source parameters are listed in **Table S2**.

A method detection limit (MDL) study was performed using reagent water fortified with the target compounds, each spiked at 5 ng/L, except for THC, THC-OH, and THC-COOH which were spiked at 50 ng/L. MDLs were calculated with the appropriate student's t-value (n=12), and method reporting limits (MRLs) were set at approximately 3-5 times the MDL and adjusted for the dilution factor. Target compounds, MRLs, and multiple reaction monitoring (MRM) transitions are listed in **Table S1**. Calibration curves were made fresh for each analysis by spiking concentrated stock solutions (50/100 µg/L in methanol) accordingly in 10 mL of reagent water, along with isotopes. Calibration ranged from 5-1,250 ng/L, except for THC, THC-OH, and THC-COOH which ranged from 10-2,500 ng/L. All standards were purchased from Cerilliant, except for ecgonine which was purchased from Restek (Centre County, PA, USA).

Table S1. High-risk substance and metabolite compound list with associated isotopes, method reporting limits (MRLs), and multiple reaction monitoring (MRM) transitions.

Compound	Isotope	MRL (ng/L)	Q1 (m/z)	Q3 (m/z)[c]
6-Acetylmorphine	d6-6-acetylmorphine	50	328	165 (211)
Amphetamine	d8-amphetamine	100	136	91 (119)
Benzoylecgonine	d8-benzoylecgonine	50	290	168 (105, 82.3)
Cocaine	d3-cocaine	50	304	182 (82, 105)
Codeine	d6-codeine	50	300	152 (115)
EDDP	d3-EDDP	50	278	234 (186, 219)
Ecgonine	d3-EME[a]	100	186	100.3 (168)
EME	d3-EME	50	200	82 (182)
Fentanyl[b]	d5-fentanyl	50	337	188 (105)
Heroin	d9-heroin	100	370	165 (268)
Hydrocodone	d6-hydrocodone	50	300	199 (128)
MDA	d5-MDA	100	180	105 (133, 77)
MDMA	d5-MDMA	100	194	163 (135, 77)
Methadone	d9-methadone	50	310	105 (265)
Methamphetamine	d8-methamphetamine	100	150	91 (119)
Morphine	d6-morphine	50	286	152 (165)
Norcocaine	d3-norcocaine	50	290	168 (136)
Norfentanyl	d5-norfentanyl	50	233	84 (150)
Oxycodone	d6-oxycodone	50	316	241 (256)
THC	d3-THC	1,000	315	193 (123)
THC-COOH	d9-THC-COOH	1,000	345	193 (299)
THC-OH	d3-THC-OH	1,000	331	193 (201, 313)
Tramadol	d3-tramadol	50	264	58 (42.2)
Xylazine[b]	d6-xylazine	250	221	164 (90)

[a] d3-ecgonine is not available so ecgonine quantitation is based on the next closest isotope with respect to structure and retention time, which is d3-EME in this method.

[b] Xylazine and fentanyl were added to the analyte list in 2025.

[c] ( ) – confirmation product ions.

Abbreviations: EDDP = 2-ethylidene-1,5-dimethyl-3,3-diphenylpyrrolidine; EME = ecgonine methyl ester; MDA = 3,4-methylenedioxyamphetamine; MDMA = 3,4-methylenedioxymethamphetamine; THC = delta-9-tetrahydrocannabinol; THC-OH = 11-hydroxy-delta-9-tetrahydrocannabinol; THC-COOH = 11-nor-9-carboxy-delta-9-tetrahydrocannabinol.

Table S2. Mass spectrometer source parameters in electrospray ionization (ESI) positive mode.

Parameter	Value
Curtain gas (CUR)	20
Collision gas (CAD)	12
Ion spray voltage (IS)	5500
Ion source gas 1 (GS1)	60
Ion source gas 2 (GS2)	50
Temperature (TEM)	550
Entrance potential (EP)	10

Figure S1. Concentrations of high-risk substances by year during the festival week/weekend for the WWTP (red), Off-site (Bars, Las Vegas Strip, and Airport; green), and Festival site (blue) wastewater samples. Comparison grab samples are not shown. A comparison of festival and non-festival weeks for the WWTP and Off-site locations is provided in Figure S2.

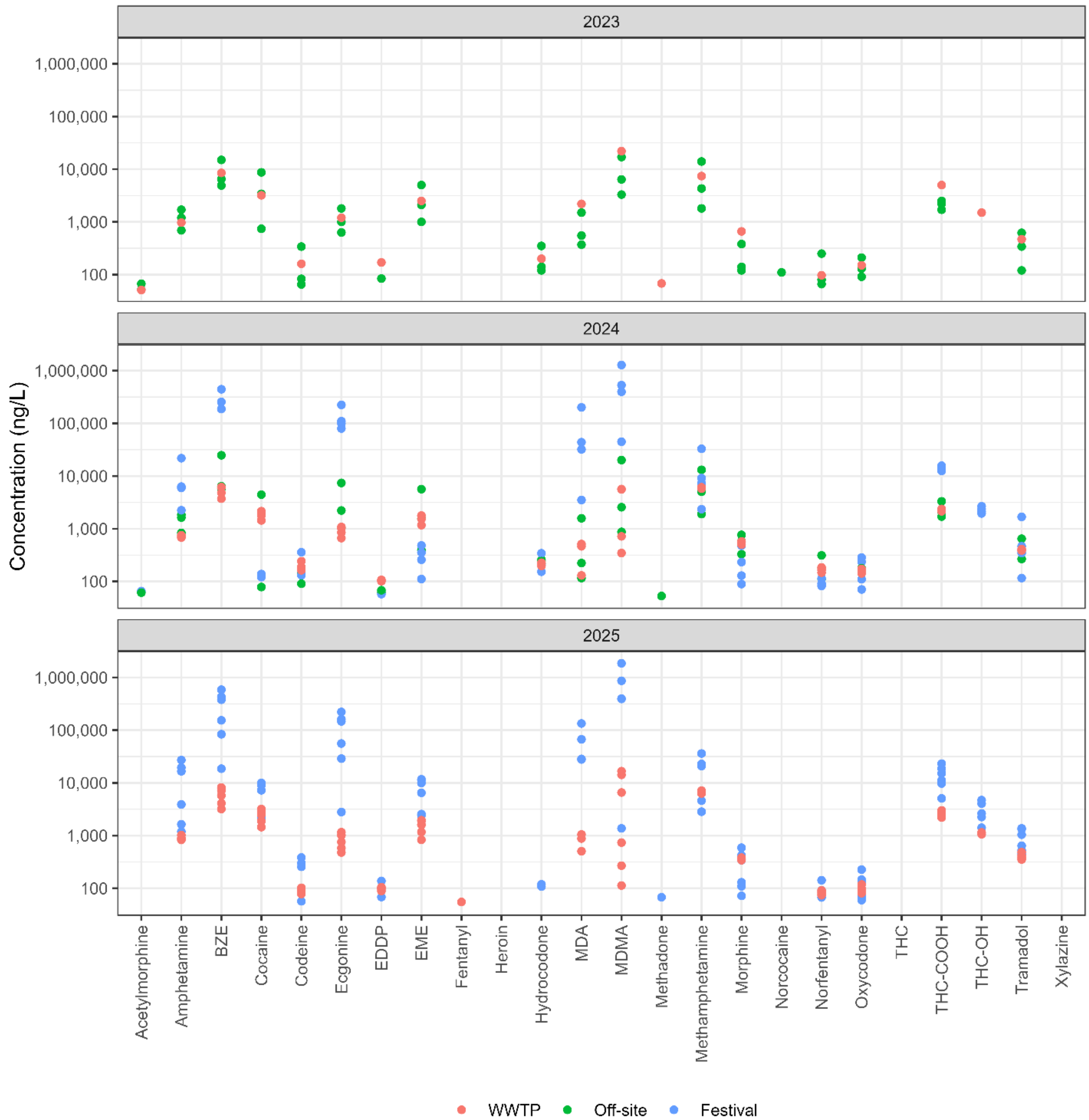

Figure S2. 2023 concentrations of high-risk substances in wastewater by month of sample collection for the Bars (red), Las Vegas Strip (green), Airport (blue), and WWTP (purple) for (A) stimulants, (B) opioids, and (C) sedatives (THC-COOH and THC-OH only). The May sampling event represents the festival weekend; the June and July sampling events were considered non-festival baseline weekends. **Note:** Non-detect analytes are omitted from this figure.

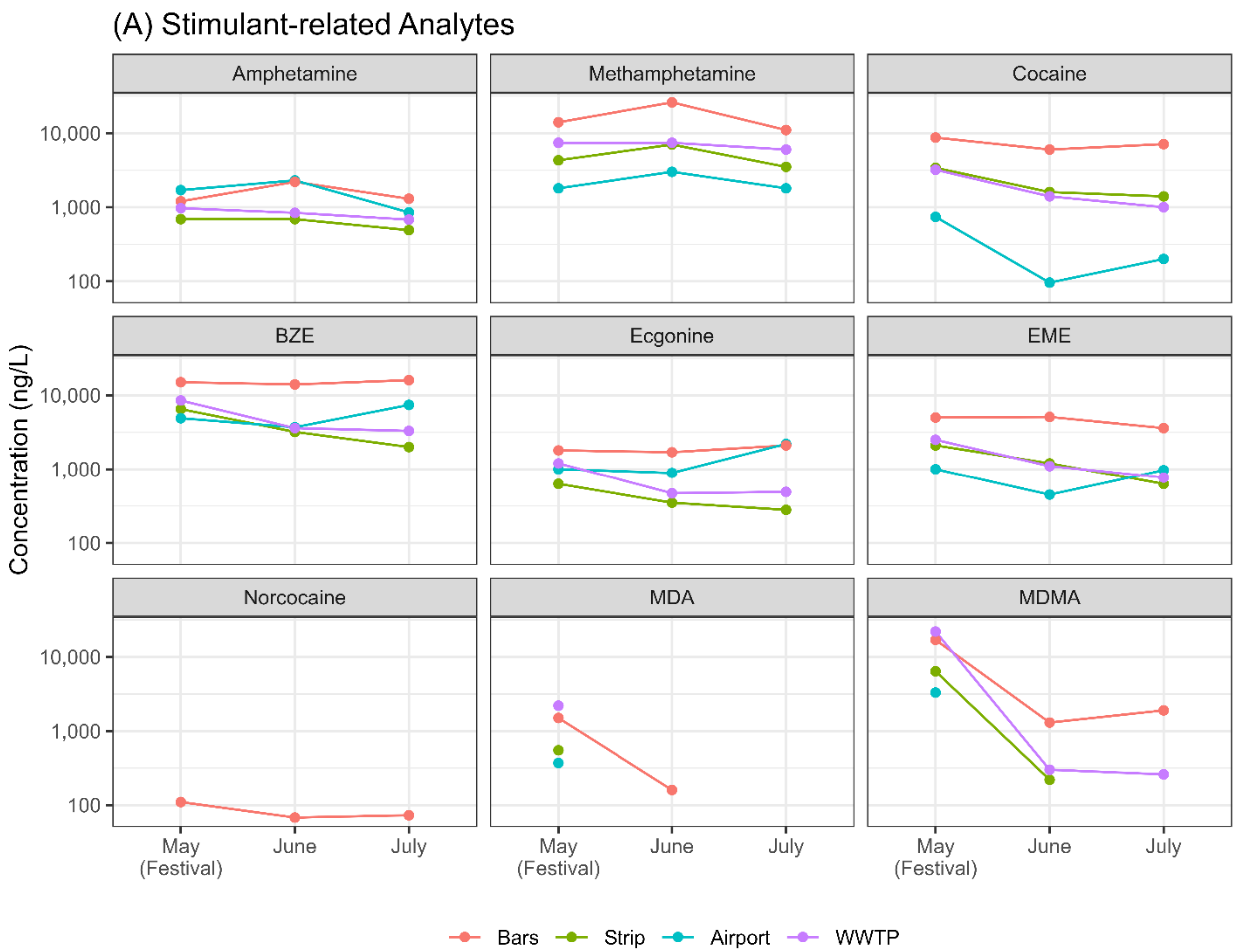

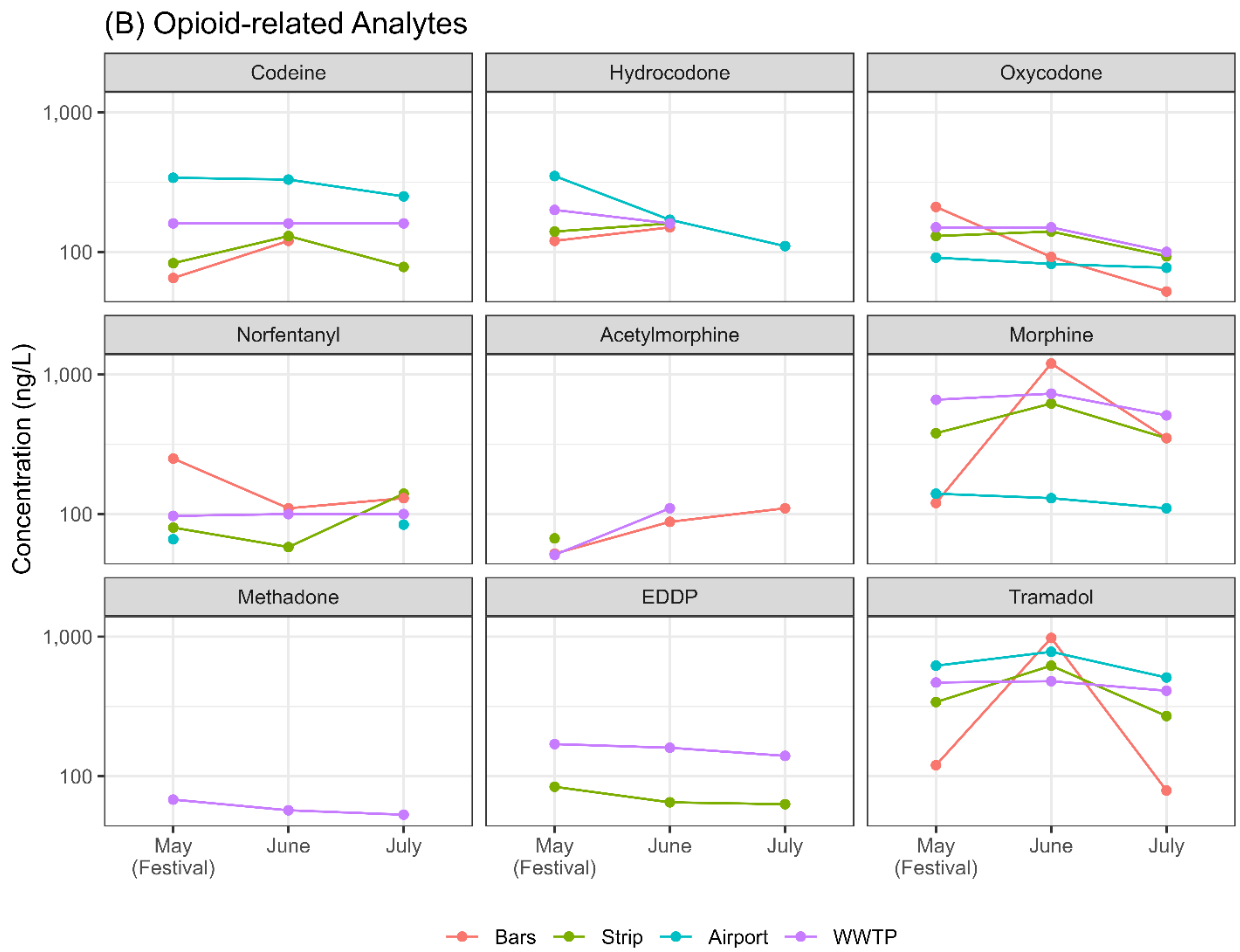
(B) Opioid-related Analytes
Codeine
Hydrocodone
Oxycodone
Norfentanyl
Acetylmorphine
Morphine
Methadone
EDDP
Tramadol
Concentration (ng/L)
1,000
100
May
(Festival)
June
July
Bars
Strip
Airport
WWTP

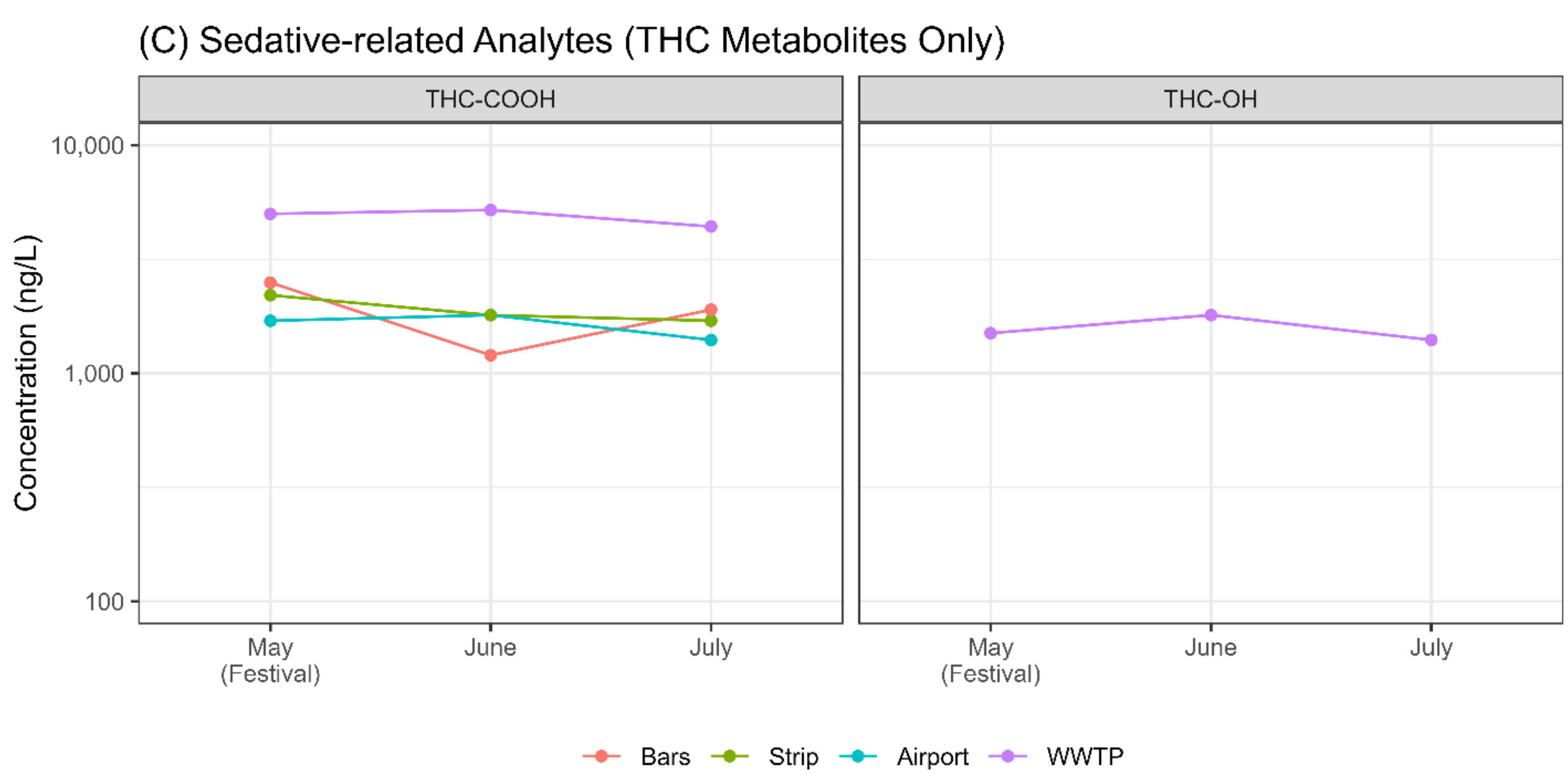
(C) Sedative-related Analytes (THC Metabolites Only)
THC-COOH
THC-OH
Concentration (ng/L)
10,000
1,000
100
May
(Festival)
June
July
Bars
Strip
Airport
WWTP

Table S3. Wastewater concentrations of analytes and corresponding sample information **[see corresponding data file (.XLSX)]**. All laboratory and field blank samples were non-detect for all analytes. <MRL = Below the method reporting limit.

Text S2. Additional discussion related to on-site and community-level public health activities.

On-site public health activities related to the music festival

By leveraging wastewater surveillance data, event organizers, safety personnel, and first responders alike can allocate resources and prioritize services more effectively based on their understanding of attendee characteristics and health risks [1]. Importantly, the festival discussed here enforces a "zero-tolerance" drug policy and minimum age requirement (18+), and attendees and their belongings are subject to search at entrance security checkpoints for the festival grounds and the campsite. Nevertheless, the wastewater data suggest that drug use is still occurring either on-site during the event or off-site prior to arriving at the event; in either case, there could be adverse public health impacts for which these special events should be prepared to address. The music festival in question ensures that related health information is readily available on the event website, in addition to on-site messaging about the risks of substance use (e.g., fentanyl awareness). First aid tents and water stations are spread throughout the festival grounds along with medics and emergency response personnel and on-site harm reduction/safety staff among the crowd to provide assistance, respond to emergencies, and support attendee safety with a "no questions asked" approach. In recent years, free naloxone has been distributed at the festival grounds and at the campsite, after attendees complete a brief training on how to use/administer it. In collaboration with public health officials, music festival organizers could leverage historical or ongoing wastewater surveillance data to seek and acquire additional funding for these preparedness efforts.

In addition to the measures above, future responses informed by wastewater results may further address stimulant use (e.g., cocaine, MDMA, methamphetamine) at festivals. Continued surveillance of drug refuse (e.g., discarded drug paraphernalia) and overdoses, including

stimulant-related overdose tracking, supports situational awareness of evolving substance use trends via public dashboards. Existing health promotion messaging channels (e.g., official health agency websites and communications, community-based outreach and educational trainings, press releases and targeted media campaigns, and public meetings and engagement with community partners) could further highlight the health risks of stimulants and polysubstance use. Extending access to drug checking supplies could help dance festival attendees identify adulterated drugs [2,3]. With expedited sample turnaround times, wastewater surveillance data could be updated on a daily basis and disseminated through event-specific or agency dashboards to raise awareness of risks associated with recreational drug use in near-real-time. These data do not necessarily have to be limited to high-risk substances, as there would also be public health value in providing near-real-time data on infectious disease targets that could potentially be monitored in the same samples. Efforts should also continue to confront factors that compound the risks of festival-related drug use, such as crowding, heat, dehydration, and exhaustion [4,5]. In response to heat-related health concerns, for example, the festival in this study has been held in May instead of June since 2018 [6].

Off-site/community-level public health activities related to the music festival

Findings from the WWTP samples and the June and July 2023 off-site, non-festival sampling events emphasize that substance use is not only associated with the festival site/weekend. HRS were detected in community wastewater throughout the 2022-2023 study in Southern Nevada, including in primarily residential as well as tourism-impacted sewersheds [7]. As in large urban centers like New York City [8], nightlife and tourist attractions and related health and substance use behaviors can occur year-round in Las Vegas and in other travel/event destinations and communities. Thus, there is an ongoing need to continue public health responses

and investment (e.g., distribution of naloxone and test strips, provision of community trainings, health education messaging and awareness campaigns, etc.) throughout the year to protect the health and safety of residents and visitors alike.

In Southern Nevada, the local public health authority (Southern Nevada Health District, SNHD) and non-profit partners invest in event-focused prevention and health promotion activities. In 2025, festival activities were accompanied by off-site distribution of harm reduction supplies (e.g., naloxone, test strips/kids) at hotels catering to festivalgoers. During the week leading up to the festival, a local non-profit event specifically welcomed festival attendees to obtain these supplies and additional health promotion items (e.g., condoms).

Supported by multiple funding sources, SNHD also collaborates with community partners, healthcare providers, and people with lived experience to implement evidence-based overdose prevention strategies throughout the year. Using data-driven approaches, SNHD operates the Linkage to Action (L2A) team in carceral and community settings to connect individuals to treatment, housing, and peer support. A post-overdose response team (SPORT) provides 24/7 follow-up care and prevention resources, complementing efforts of door-to-door canvassing, outreach to high-risk areas, and overdose prevention training. Naloxone and test strips are distributed through community partners, mail order, and vending machines/kiosks [9].

Additional health promotion efforts include agency communications (e.g., website, social media, press releases), targeted media campaigns, in-person and virtual overdose prevention and education trainings, and engagement with partner networks, including the Southern Nevada Opioid Advisory Council (SNOAC), healthcare providers, prevention coalitions, and community organizations. SNHD also maintains a public data dashboard and convenes an annual summit to strengthen coordinated overdose prevention and response efforts.

Text S3. References.

1. Hutton A, Ranse J, Munn MB. Developing Public Health Initiatives through Understanding Motivations of the Audience at Mass-Gathering Events. *Prehosp Disaster Med*. 2018;33(2):191-196. doi:10.1017/S1049023X18000067
2. Day N, Criss J, Griffiths B, et al. Music festival attendees' illicit drug use, knowledge and practices regarding drug content and purity: a cross-sectional survey. *Harm Reduct J*. 2018;15(1):1. doi:10.1186/s12954-017-0205-7
3. Palamar JJ, Fitzgerald ND, Keyes KM, Cottler LB. Drug Checking at Dance Festivals: A Review With Recommendations to Increase Generalizability of Findings. *Exp Clin Psychopharmacol*. 2021;29(3):229-235. doi:10.1037/pha0000452
4. Palamar JJ, Sönmez İ. A qualitative investigation exploring why dance festivals are risky environments for drug use and potential adverse outcomes. *Harm Reduct J*. 2022;19(1):12. doi:10.1186/s12954-022-00598-5
5. Polkinghorne BG, Massey PD, Durrheim DN, Byrnes T, MacIntyre CR. Prevention and surveillance of public health risks during extended mass gatherings in rural areas: The experience of the Tamworth Country Music Festival, Australia. *Public Health*. 2013;127(1):32-38. doi:10.1016/j.puhe.2012.09.014
6. Brown A. Pasquale Rotella talks heat-related EDC date change: 'It wasn't as comfortable as it needed to be.' *Los Angeles Times*. September 28, 2017.
7. Gerrity D, Crank K, Oh EC, Quinones O, Trenholm RA, Vanderford BJ. Wastewater surveillance of high risk substances in Southern Nevada: Sucralose normalization to translate data for potential public health action. *Science of The Total Environment*. 2024;908:168369. doi:10.1016/j.scitotenv.2023.168369
8. Palamar JJ, Le A, Cleland CM, Keyes KM. Trends in drug use among nightclub and festival attendees in New York City, 2017-2022. *International Journal of Drug Policy*. 2023;115:104001. doi:10.1016/j.drugpo.2023.104001
9. Allen ST, O'Rourke A, Johnson JessicaA, et al. Evaluating the impact of naloxone dispensation at public health vending machines in Clark County, Nevada. *Ann Med*. 2022;54(1):2680-2688. doi:10.1080/07853890.2022.2121418
